# Early Intrableb Features on Anterior Segment Swept-Source Optical Coherence Tomography Predict Surgical Success After Trabeculectomy in Uveitic and Neovascular Glaucoma

**DOI:** 10.3390/jcm14155499

**Published:** 2025-08-05

**Authors:** Sangwoo Moon, Seungmin Lee, Jiwoong Lee

**Affiliations:** 1 Department of Ophthalmology, Pusan National University Yangsan Hospital, Pusan National University School of Medicine and Research Institute for Convergence of Biomedical Science and Technology, Yangsan 50612, Republic of Korea; platinummetal@hanmail.net; 2 Department of Ophthalmology, Pusan National University Hospital, Pusan National University School of Medicine and Biomedical Research Institute, Busan 49241, Republic of Korea; glaucoma@pusan.ac.kr

**Keywords:** early filtering bleb, anterior segment swept-source optical coherence tomography, uveitic glaucoma, neovascular glaucoma

## Abstract

**Background**: This study aimed to evaluate prognostic factors of early filtering blebs using anterior segment swept-source optical coherence tomography (AS SS-OCT) in patients with uveitic and neovascular glaucoma. **Methods**: This retrospective cohort study included 22 eyes from 22 patients who underwent trabeculectomy (11 eyes each with uveitic or neovascular glaucoma). Intrableb characteristics were assessed using AS SS-OCT at 1 month, postoperatively. Surgical success was defined as intraocular pressure (IOP) ≤ 18 mmHg and ≥30% IOP reduction without medication at 12 months. Logistic regression was used to identify the prognostic factors associated with IOP control. **Results**: Sixteen eyes (72.7%) achieved surgical success, while six (27.3%) were unsuccessful. Eyes with successful IOP control at 12 months showed thicker and less reflective bleb walls with microcysts compared with unsuccessful cases of IOP control, in the early postoperative phase (all *p* < 0.033). However, IOP at the time of OCT did not significantly differ between the groups (*p* = 0.083). Multivariate logistic regression analysis revealed that higher bleb wall reflectivity at 1-month post-trabeculectomy was significantly associated with a higher surgical failure rate at 12 months after trabeculectomy (hazard ratio = 1.072, *p* = 0.032). **Conclusions**: Early intrableb assessment using AS SS-OCT may be beneficial for managing filtering blebs after trabeculectomy in uveitic and neovascular glaucoma. Higher bleb wall reflectivity in the early post-trabeculectomy phase may indicate poor features of the filtering bleb, suggesting the need for timely interventions for refractory cases.

## 1. Introduction

Glaucoma is a leading ophthalmic disease that can cause irreversible blindness [1]. In particular, refractory glaucoma, including neovascular glaucoma (NVG) or uveitic glaucoma (UG), often requires surgical intervention because approximately 50% of NVG cases and 30% of UG cases do not respond adequately to conventional medical treatments [2,3].

Trabeculectomy is a standard surgical procedure for managing these conditions; however, its outcomes are often suboptimal. Reported one-year success rates range from 50% to 75% in NVG and approximately 71.3% in UG, which are lower than those typically observed in primary open-angle glaucoma (POAG) [3,4]. Studies investigating the etiology of NVG and UG associated with surgical failure of trabeculectomy have shown inconsistent results [5,6,7,8].

Nonetheless, several reports suggest that postoperative complications, including prolonged postoperative inflammation, hypotony, bleb leak, hyphema, persistent neovascularization, vitreous hemorrhage, and chronic granulomatous inflammation, significantly diminish trabeculectomy success rates in patients with NVG or UG [9,10,11]. These complications are believed to contribute to scarring of the surgical site, subsequently reducing aqueous humor flow from the anterior chamber into the subconjunctival space [3,4,12]. Excessive subconjunctival fibrosis is the most common cause of surgical failure, as it prevents or limits egress of the aqueous humor [13].

Previous studies have emphasized that early changes in filtration bleb morphology can significantly influence long-term surgical outcomes, underscoring the importance of proactive postoperative management [12,14]. Several imaging modalities have been developed to evaluate the morphology and function of filtration blebs [12,14,15,16,17]. Among them, anterior segment optical coherence tomography (AS-OCT) has emerged as a valuable tool for precise intrableb structural analysis [12,14,16,17]. A recent meta-analysis reported that a greater bleb height, thicker bleb wall, and lower reflectivity on AS-OCT were consistently associated with successful trabeculectomy outcomes [18].

However, conventional spectral-domain OCT is limited by a shallow penetration depth, which restricts the evaluation of deeper bleb structures [16,17]. In contrast, anterior segment swept-source OCT (AS SS-OCT) offers a deeper penetration depth of over 10 mm, enabling more comprehensive structural assessments [19].

This study aimed to analyze early postoperative filtration bleb changes that influence surgical outcomes in patients with uveitic and neovascular glaucoma. Using AS SS-OCT for detailed assessment, we sought to elucidate the relationship between early bleb morphology and surgical success, with the goal of informing strategies for enhanced postoperative management and long-term surgical outcomes.

## 2. Materials and Methods

### 2.1. Ethics Statement

This study was conducted in accordance with the tenets of the Declaration of Helsinki and was approved by the Institutional Review Board of Pusan National University Yangsan Hospital (No. 55-2024-076). Written informed consent was obtained from all patients for the surgical procedures and for the storage of their information in the hospital database as well as its use for research purposes.

### 2.2. Study Design

This retrospective cohort study included patients diagnosed with UG or NVG who underwent fornix-based trabeculectomy with 0.04% mitomycin C (MMC) between March 2023 and February 2024 at the Department of Ophthalmology at Pusan National University Yangsan Hospital (Yangsan, Republic of Korea). All patients were observed for a minimum duration of 12 months after surgery. Only the first operated eye was included in the analysis if both eyes underwent trabeculectomy.

“Refractory glaucoma” was defined as eyes with a history of prior failed trabeculectomy, neovascular glaucoma, or uveitic glaucoma [20]. In this study, patients with neovascular or uveitic glaucoma had not undergone any previous incisional glaucoma surgeries. Surgical indications included (1) inadequate intraocular pressure (IOP) control despite maximally tolerated medical therapy and (2) intolerance or allergy to glaucoma medications. Surgery for UG is generally deferred until inflammation has been quiescent for at least three months, unless urgent intervention is needed due to severe IOP elevation threatening the optic nerve. In NVG, surgery is typically performed at least 48 h after an intravitreal anti-VEGF injection, once neovascular regression is confirmed.

Before surgery, all participants underwent full ophthalmic evaluations, including best-corrected visual acuity testing, slit-lamp biomicroscopy, IOP measurement via Goldmann applanation tonometry, gonioscopy, a dilated fundus examination, a stereoscopic optic disk assessment, and red-free retinal nerve fiber layer photography (AFC-210; Nidek, Aichi, Japan). Biometric data were obtained using the IOLMaster (Carl Zeiss Meditec, Dublin, CA, USA), and standard automated perimetry was also conducted. Central corneal thickness was measured with ultrasonic pachymetry (Pachmate; DGH Technology, Exton, PA, USA) and keratometry was assessed using an Auto Kerato-Refractometer (ARK-510A; NIDEK, Aichi, Japan).

### 2.3. Surgical Technique

Trabeculectomy with 0.04% MMC was performed under local anesthesia by a single surgeon (S.M.). In the superonasal quadrant, a 6 mm limbal conjunctival incision was made to create a fornix-based conjunctival flap, followed by careful dissection of the conjunctiva and Tenon’s capsule towards the conjunctival sac. A trapezoidal scleral flap was also made, measuring 4.5 mm at the base, 3.0 mm at the apex, and 3.0 mm on each side, representing approximately half of the scleral thickness. Surgical sponges (Eye Spear, Huizhou Foryou Medical Devices Co., Huizhou, China), soaked in 0.4 mg/mL (0.04%) MMC, were placed between Tenon’s capsule and the sclera for 3.0 min. After removing the sponge, the exposed area was thoroughly irrigated with 20 mL of a balanced salt solution (BSS). Inner sclerostomy and peripheral iridectomy were performed. The scleral flap was secured using two preplaced 9-0 nylon (Ethicon Inc., Johnson & Johnson, Somerville, NJ, USA) releasable sutures. The conjunctiva and Tenon’s capsule were repositioned anteriorly and secured using interrupted 9-0 nylon sutures. The anterior chamber was reformed with a BSS, and the patency of the scleral flap and the absence of bleb leakage at the conjunctival closure site were confirmed.

After surgery, patients received topical moxifloxacin (Moroxacin^®^, Hanmi Pharm, Co., Seoul, Republic of Korea) four times daily and prednisolone acetate (Pred forte^®^, AbbVie Inc, North Chicago, IL, USA) six times daily for one month, followed by a gradual taper over 12 weeks based on bleb morphology and IOP. Steroids were continued in cases where postoperative inflammation was not well controlled or neovascularization recurred. Bleb management, including digital massage or bleb needling, was performed when bleb function was deemed inadequate during the follow-up. Bleb morphology was assessed postoperatively at 1 month using the Indiana Bleb Appearance Grading Scale [21].

### 2.4. Definition of Surgical Success

Surgical success was defined as an IOP of ≤18 mm Hg with a reduction of ≥30% from baseline, without the use of glaucoma medications, at 12 months, postoperatively [22,23]. If the baseline IOP was ≤18 mm Hg, a reduction of ≥30% from baseline was considered a surgical success. Eyes were additionally considered to have achieved surgical success only if there were no serious complications, such as loss of light perception or the need for additional glaucoma surgery (including repeat trabeculectomy or tube shunt implantation), during the follow-up period.

### 2.5. AS SS-OCT Imaging

All patients underwent AS SS-OCT 1 month after trabeculectomy. Postoperative blebs were imaged using CASIA2 (Tomey Corporation, Nagoya, Japan), which utilizes a 1310 nm swept-source laser wavelength and a scanning speed of 50,000 A-scans per second. The standard settings of the “Bleb” imaging mode (scan pattern size 12 × 12 mm raster scan, 256 B-scans, 400 A-scans per B-scan) were used. The device had a penetration depth of up to 14 mm, a transverse resolution of ≤30 μm and an axial resolution of ≤10 μm. The structural parameters of bleb were measured using the device’s built-in software (Version: 4B.2), while bleb wall reflectivity was analyzed using the ImageJ software (ImageJ 1.50b, http://imagej.nih.gov/ij/; developed by Wayne Rasband, National Institutes of Health, Bethesda, MD, USA) [24].

**Figure 1:**
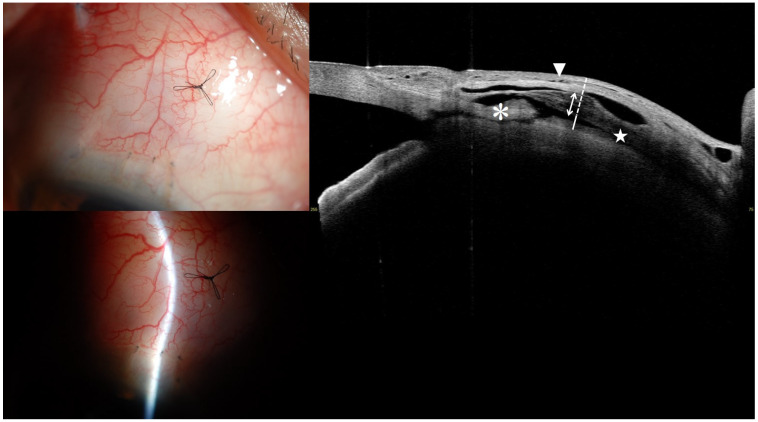
Anterior segment swept-source optical coherence tomography image for measuring intrableb parameters. The white and white dotted line indicate the fluid-filled space height and bleb wall thickness, respectively. The white two-way arrow represents the hyporeflective layers with striping phenomenon. The star denotes the fluid-filled space, while the asterisk indicates the scleral flap. The white arrowhead points to the microcyst.

At the point of maximum bleb elevation, horizontal (tangential to the limbus) and vertical (radial and perpendicular to the limbus) scans were acquired. Quantitative measurements included maximum bleb height, bleb wall thickness, stripping layer thickness, the ratio of stripping layer to bleb wall thickness, fluid-filled space height and area, and bleb wall reflectivity (Figure 1). The mean of the horizontal and vertical scan values was used for analysis. The quantitative parameters are defined as follows (Figure 1):

Bleb height was assessed by measuring the maximum vertical distance from the first reflective signal of the conjunctiva, following a line orthogonal to the scleral curvature (Figure 1) [16,17]. Bleb wall thickness was measured as the greatest perpendicular distance extending from the conjunctival reflective layer to the peak of the underlying fluid-filled cavity, encompassing both the conjunctiva and Tenon’s capsule (Figure 1) [16,17]. Within Tenon’s capsule, the striping layer is identified by multiple aligned, fluid-filled channels that create a honeycomb-like architecture (Figure 1) [25,26].

The height of the fluid-filled space was evaluated by determining the maximum vertical span within the hyporeflective or signal-absent area between the inner bleb wall base and the scleral apex, measured along a line orthogonal to the scleral tangent (Figure 1) [16,17]. The fluid-filled space area refers to the largest measured region of the signal-absent or hyporeflective zone between the inner bleb wall base and the apex of the sclera [16,17].

Bleb wall reflectivity was assessed by placing elliptical markers at three equidistant points (anterior, middle, and posterior) along the bleb wall and adjacent background. The background reflectivity was subtracted from each corresponding bleb wall point, and the mean reflectivity value of the bleb wall was utilized in the analysis [24].

Microcyst formation was assessed qualitatively and described as the existence of hyporeflective or signal-absent areas situated within or just beneath the epithelial layer of the bleb wall [14].

### 2.6. Statistical Analysis

All statistical analyses were performed using IBM SPSS ver. 26.0 (IBM Corp., Armonk, NY, USA). The normality of the data distribution was assessed using the Kolmogorov–Smirnov test. Differences between the two groups categorized by surgical success were evaluated using either the Mann–Whitney U-test or independent-sample *t*-test for continuous variables, and the Chi-squared or Fisher’s exact test for categorical variables.

Multivariate logistic regression analysis with stepwise forward selection was used to identify AS SS-OCT-based intrableb structural parameters that were significantly associated with successful IOP control. The odds ratios (ORs) for these parameters were calculated. Independent variables included sex, glaucoma type (NVG or UG), and preoperative lens status as categorical variables. Continuous independent variables included age, number of preoperative glaucoma medications, axial length, preoperative visual acuity, preoperative mean deviation, preoperative IOP, and postoperative IOP at 1 month. The intrableb parameters analyzed as independent variables were bleb height, bleb wall thickness, striping layer thickness, striping-to-bleb wall ratio, bleb wall reflectivity, fluid-filled space height and area, and presence of microcysts. Variables with *p* < 0.20 in univariable analysis were included in the multivariable analysis. *p* < 0.05 was considered statistically significant.

## 3. Results

### 3.1. Demographics and Clinical Characteristics in All Patients

**Figure 2:**
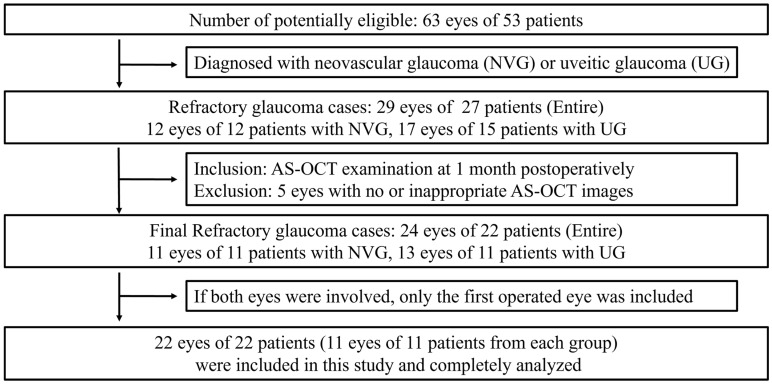
Flow diagram demonstrating patient selection in each group.

A total of 63 eyes from 53 patients underwent trabeculectomy between March 2023 and February 2024, and 29 eyes from 27 patients were diagnosed with refractory glaucoma (12 eyes of 12 patients with NVG and 17 eyes of 15 patients with UG). Among these, 5 eyes were excluded owing to no or inappropriate AS SS-OCT images at 1 month after surgery. In patients with UG, only the first operated eye was included because both eyes were involved in two cases. Finally, a total of 22 eyes from 22 patients were enrolled in this study, with each group comprising 11 eyes from 11 patients (Figure 2). Table 1 provides a summary of the demographic and clinical features of the patients in each group. The overall frequency of successful IOP control at one year was 72.7% (16/22). There were two and four unsuccessful cases for UG and NVG, respectively. In this study, almost all cases, except one with an encapsulated bleb in the unsuccessful group, had clinically diffuse and healthy blebs without any signs of an encapsulated bleb (all *p* ≥ 0.094) (IBAGS, Table 2). All uveitis cases were limited to the anterior chamber, and inflammation was well controlled in all patients for at least three months prior to trabeculectomy. In this study, VZV and CMV were detected by anterior chamber polymerase chain reaction in two patients, respectively. Only one patient, who was diagnosed with Behcet disease, was on systemic medications including colchicine (0.6 mg once daily), azathioprine (75 mg once daily). All patients with NVG underwent panretinal photocoagulation, and those with accompanying macular edema (seven with PDR, two with CRVO) received additional anti-VEGF or intravitreal steroid treatment by a retina specialist.

### 3.2. Comparison of Anterior Segment Optical Coherence Tomography Parameters at 1 Month After Trabeculectomy

The early intrableb structures assessed using AS SS-OCT at 1 month, postoperatively, were compared between the two groups based on surgical success at 1 year (Table 3). In terms of early intrableb structures, eyes with successful IOP control exhibited a thicker bleb wall (*p* = 0.033), lower bleb wall reflectivity (*p* = 0.021), and more microcyst formation (*p* = 0.025) than eyes with unsuccessful IOP control. However, no significant differences were observed in bleb height, stripping layer thickness, striping-to-bleb wall ratio, or fluid-filled space area and height (All *p* ≥ 0.083).

### 3.3. Logistic Regression Model of the Variables Associated with Successful IOP Control Postoperatively at 1 Year

In the univariate analysis, higher bleb wall reflectivity (*p* = 0.032) and the absence of microcyst formation (*p* = 0.022) at 1 month were significantly associated with surgical failure at 1 year. The difference in postoperative IOP at 1 month was not statistically significant (*p* = 0.083). Other variables with *p* < 0.20 included age, bleb height, and bleb wall thickness. These variables—bleb wall reflectivity, microcyst formation, postoperative IOP at 1 month, age, bleb height, and bleb wall thickness—were included in the multivariate analysis. Among them, only bleb wall reflectivity at 1 month was significantly associated with a surgical failure rate (hazard ratio, 1.072; confidence interval, 1.006–1.141; *p* = 0.032) (Table 4). There were no significant differences in early intrableb parameters between the two successful groups (All *p* ≥ 0.351) (Supplementary Table S1).

### 3.4. Representative Anterior Segment Optical Coherence Tomography Images at 1 Month

**Figure 3:**
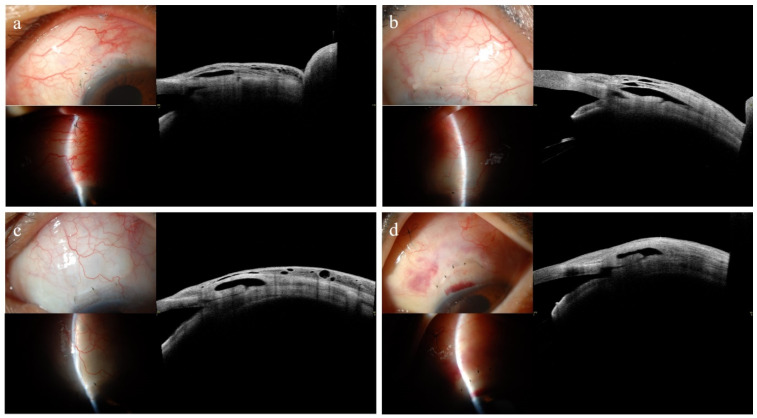
Representative AS-OCT images of filtering blebs at 1-month post-trabeculectomy in eyes with different surgical outcomes at 1 year. Images in the left column represent the successful group, while those in the right column represent the unsuccessful group. All blebs appeared clinically diffuse, without signs of encapsulation on slit-lamp examination. (**a**) A 61-year-old man with uveitic glaucoma (UG) in the left eye showed an intraocular pressure (IOP) of 10 mmHg. The bleb exhibited a prominent striping layer, diffuse microcyst formation, and low bleb wall reflectivity. (**b**) A 63-year-old man with UG in the right eye had an IOP of 14 mmHg. The bleb showed a larger fluid-filled space but less striping, limited anterior microcysts, and higher reflectivity. (**c**) A 56-year-old man with neovascular glaucoma (NVG) in the left eye achieved an IOP of 8 mmHg with a bleb showing limited fluid-filled space, diffuse microcyst formation, and low reflectivity. (**d**) A 50-year-old woman with NVG in the left eye had an IOP of 10 mmHg. Despite a similar fluid-filled space to (**c**), the bleb demonstrated higher reflectivity, absence of striping, and minimal microcyst formation.

Figure 3 illustrates the differences in bleb wall reflectivity at 1 month, postoperatively, in patients who underwent trabeculectomy according to the surgical success at 12 months, postoperatively. All blebs appeared clinically diffuse and healthy, without any signs of encapsulation in the slit-lamp photographs. As shown in Figure 3a (successful group), a 61-year-old man diagnosed with UG underwent trabeculectomy in the left eye and maintained an IOP of 10 mmHg without the need for glaucoma medications. The bleb exhibits a prominent striped layer, evident microcyst formation, and low bleb wall reflectivity. In Figure 3b (unsuccessful group), a 63-year-old man diagnosed with UG and treated with trabeculectomy in the right eye had an IOP of 14 mmHg without glaucoma medications. Although the bleb showed a greater fluid-filled space than that shown in Figure 3a, it exhibited a smaller stripping layer and a higher bleb wall reflectivity. Microcyst formation was limited to the anterior portion of the bleb wall. In Figure 3c (successful group), a 56-year-old man with NVG underwent trabeculectomy in the left eye and exhibited an IOP of 8 mmHg without glaucoma medication. Despite the limited fluid-filled space, the bleb demonstrated diffuse microcyst formation and low bleb wall reflectivity. As shown in Figure 3d (unsuccessful group), a 50-year-old woman with NVG, who underwent trabeculectomy in the left eye, had an IOP of 10 mmHg without glaucoma medications. Although the fluid-filled space was similar to that shown in Figure 3c, the bleb exhibited a higher bleb wall reflectivity, lacked a striping layer, and exhibited minimal or no microcyst formation.

## 4. Discussion

In the present study, we evaluated distinctive early intrableb characteristics associated with successful trabeculectomy at one year using AS SS-OCT in patients diagnosed with neovascular or uveitic glaucoma. To the best of our knowledge, this is the first study to evaluate the intrableb structures using AS SS-OCT after fornix-based trabeculectomy in patients with uveitic and neovascular glaucoma.

Our findings revealed significantly different early intrableb structures at one month postoperatively between the successful and unsuccessful groups at 1 year, postoperatively. Although there were no significant differences in bleb morphology based on the IBAGS, blebs in the successful group had significantly thicker bleb walls, lower bleb wall reflectivity, and more prominent microcyst formation than those in the unsuccessful group. Multivariate logistic regression analysis confirmed that only a lower bleb wall reflectivity at 1 month, postoperatively, was significantly associated with surgical success at 1 year. The findings of the present study are consistent with those of previous studies.

In our previous studies and a recent meta-analysis, blebs with successful trabeculectomy outcomes showed common features of greater bleb height, thicker bleb wall and striping layer, lower bleb wall reflectivity, and more frequent microcyst formation on AS-OCT [16,17,18]. Prior studies have also demonstrated that early intrableb structures are closely associated with long-term IOP control after trabeculectomy. Narita et al. [14] reported that multiple parallel hyporeflective layers (striping layers) within the bleb wall at 2 weeks after trabeculectomy were associated with good IOP control at 1 year, postoperatively. However, the bleb height, bleb wall thickness, microcyst formation, and ratio of hyporeflective space of bleb wall were not significant in the multivariate logistic regression model [14]. A time-domain AS-OCT study by Nakano et al. [27] suggested that the presence of filtering blebs with multilayered structures at 2 weeks post-trabeculectomy was associated with better bleb function at 6 months, postoperatively. Theelen et al. [26] found that the successful filtering of blebs, evaluated using slit-lamp-adapted OCT in the first postoperative week, showed a characteristic striping pattern, which was absent in unsuccessful cases. This pattern was interpreted as fluid-filled channels separated by fine connective tissue, suggesting its potential as an early marker of surgical success [26]. With these results, the authors of the present study hypothesize that these multiple parallel hyporeflective layers contribute to lower bleb wall reflectivity.

The bleb wall plays a crucial role in aqueous absorption and IOP control. Because the aqueous humor inside a functioning bleb passes through the conjunctiva, where it either mixes with the tear film or is absorbed by nearby vascular, perivascular conjunctival tissues, and lymphatic vessels adjacent to the surgical site [28]. In the current study, the bleb wall-related variables including thicker bleb wall, lower bleb wall reflectivity, and more prominent microcyst formation at 1 month were associated with successful IOP control at 1 year, postoperatively. Our previous study also showed that functioning blebs had a thicker bleb wall and striping layer, lower bleb wall reflectivity, and more frequent microcyst formation than non-functioning blebs [16,17]. Histopathological and in vivo confocal microscopy studies have shown that the subepithelial connective tissue is loosely arranged in functioning blebs [29,30]. These findings likely correspond to the low bleb wall reflectivity and striping layers observed in the present study and in other AS-OCT studies [14,16,17,26,27]. In contrast, failed blebs have been reported to exhibit dense collagenous connective tissue within their walls and may show high bleb wall reflectivity [29].

NVG and UG have been reported to have poor surgical outcomes, with excessive subconjunctival fibrosis recognized as the most common cause of surgical failure, as it prevents or limits the egress of aqueous humor [2,3,13]. In NVG, the conjunctiva is often clinically inflamed in eye with NVG, making it highly primed for an aggressive postoperative wound healing response [31]. A prospective study demonstrated that eyes with UG undergoing filtration surgery exhibited increased conjunctival fibroblasts, macrophages, and lymphocytes in both the superficial and deep conjunctival stroma compared with controls, suggesting that this inflammatory cellular profile may contribute to the higher failure rate of trabeculectomy in UG [32].

However, early changes in the bleb wall are often difficult to detect using slit-lamp biomicroscopy. In the present study, although there was no significant difference in the early postoperative IBAGS at 1 month, AS SS-OCT revealed distinct differences in bleb morphology. Notably, bleb wall reflectivity, an indicator of bleb wall fibrosis, is associated with surgical success. These findings are consistent with those of previous studies and underscore the clinical value of AS-OCT in the postoperative assessment of filtering blebs. Waibel et al. [12] used AS-OCT measurements to evaluate bleb parameters such as bleb wall thickness and bleb height. Encapsulation was first indicated by a decrease in bleb wall thickness at 1 week and an increase in bleb height at 2 weeks—both preceding clinical suspicion—while IOP elevation began at 3 weeks, with encapsulation diagnosed in 4 cases at 3 weeks and 5 cases at 4 weeks [12]. Based on our present and previous studies, AS-OCT has shown an advantage in detecting signs of encapsulation much earlier than clinical evaluation.

Given the greater efficacy of early bleb management, AS-OCT–based identification of early bleb changes could support prompt and targeted intervention by glaucoma surgeons after trabeculectomy. In line with prior research and our results, early evaluation of intrableb structures using AS-OCT—particularly assessing parameters such as bleb wall reflectivity, bleb wall thickness, and microcyst presence—in patients with refractory glaucoma may facilitate postoperative bleb management, as early structural changes can serve as indicators of long-term surgical outcomes.

This study has several limitations. First, as this was a retrospective cohort study, only a small number of patients with unsuccessful IOP control were included (six eyes); therefore, the findings of this study should be interpreted with caution. Second, we were unable to compare AS SS-OCT images between one month and one year, postoperatively, because AS SS-OCT examination was not performed one year after surgery. Long-term follow-up periods may affect intrableb parameters, as described in the previous study by Narita et al. [14] For future research, we plan to evaluate OCT images at both early and late stages after trabeculectomy, as performed in the prior study [14]. Third, Although uveitic glaucoma typically presents in the fourth or fifth decade of life, patients who underwent surgery for uveitic glaucoma in this study were relatively older. In the future, it is necessary to investigate the age and clinical course of patients with uveitic glaucoma who undergo surgery versus those managed with medical therapy. Finally, it is important to note that all participants in this study were of Korean ethnicity; therefore, early postoperative changes in intrableb structures observed after trabeculectomy may not be generalizable to other populations.

## 5. Conclusions

Early evaluation of intrableb structures using AS SS-OCT may help predict surgical outcomes after trabeculectomy in patients with uveitic and neovascular glaucoma. Lower bleb wall reflectivity at one month was associated with successful IOP control at one year. AS SS-OCT detected early signs of bleb wall fibrosis before clinical suspicion, emphasizing its value for timely postoperative management. In particular, early intrableb evaluation may guide appropriate intervention because higher bleb wall reflectivity in the early postoperative phase could indicate poor bleb function. These findings support the use of AS SS-OCT as a practical tool for optimizing long-term outcomes in patients with uveitic and neovascular glaucoma.

## Figures and Tables

**Table 1:** Demographics and clinical characteristics of patients.

	Entire	Uveitic Glaucoma	Neovascular Glaucoma
Number of eyes (patients)	22 (22)	11 (11)	11 (11)
Age at trabeculectomy, years	68.62 ± 7.41	71.13 ± 5.34	66.11 ± 8.54
Sex, female	7 (31.8)	3 (27.3)	4 (36.4)
Eye laterality, right	13 (59.1)	6 (54.5)	7 (63.6)
Diagnosis			
Uveitic glaucoma	11 (50.0)	VZV (1), CMV(1), Behcet disease (1) Not definitely established (8)	PDR (9), CRVO (2)
Neovascular glaucoma	11 (50.0)		
Anterior chamber angle status, open	12 (54.55)	8 (72.7)	4 (36.4)
IOP at AS-OCT test, mmHg	9.87 ± 2.57 (6–15)	9.82 ± 2.75 (6–15)	9.91 ± 2.51 (6–15)
Preoperative visual acuity, logMAR	1.46 ± 0.96 (3.0–0)	0.89 ± 0.65 (2.0–0)	2.04 ± 0.90 (3.0–0.5)
Preoperative IOP, mmHg	32.86 ± 12.54 (15–70)	31.45 ± 8.99 (17–45)	34.27 ± 15.65 (15–70)
Number of glaucoma medication	3.86 ± 0.56 (3–5)	4.00 ± 0.63 (3–5)	3.73 ± 0.47 (3–4)
History of glaucoma medication, year	1.12 ± 1.47 (0.01–5.02)	1.35 ± 1.82 (0.01–5.02)	0.91 ± 1.11 (0.02–3.41)
Preoperative lens status, phakic	10 (45.5)	7 (63.63)	3 (27.3)
Central corneal thickness, μm	547.41 ± 34.61	553.27 ± 37.21	541.55 ± 32.49
Axial length, mm	24.27 ± 2.13	25.12 ± 2.67	23.42 ± 0.87
Spherical equivalent, diopters	−1.16 ± 1.95	−1.58 ± 1.96	−0.74 ± 1.94
Cup to disk ratio	0.81 ± 0.14	0.81 ± 0.18	0.80 ± 0.10
Visual field parameter			
Visual Field Index, %	35.55 ± 31.57	28.27 ± 28.39	42.82 ± 34.21
Mean deviation, dB	−21.73 ± 7.36	−22.97 ± 6.74	−20.49 ± 8.05
Pattern standard deviation, dB	7.39 ± 3.06	7.56 ± 3.39	7.22 ± 2.85
Successful IOP control at 1 year, %	16 (72.7)	9 (81.8)	7 (63.6)
Subsequent bleb management			
Bleb needling	8 (36.4)	2 (18.2)	6 (54.5)
1	4	2	2
≥2	4	0	4
Additional glaucoma surgery	3 (13.6)	0	3 (Trab1, AGV2)

Counting fingers at a distance of 30 cm was regarded as equivalent to a Snellen acuity of 20/2000, corresponding to a logMAR value of 2.0. Hand motion vision was considered comparable to a Snellen acuity of 20/20,000, with a logMAR of 3.0. Values are presented as mean ± standard deviation (range) or number (%) unless otherwise indicated. AGV, Ahmed glaucoma valve implantation; AS-OCT, anterior segment-optical coherence tomography; CMV, cytomegalovirus; CRVO, central retinal vein occlusion; IOP, intraocular pressure; logMAR, logarithm of the minimum angle of resolution; PDR, proliferative diabetic retinopathy; Trab, trabeculectomy; VZV, varicella zoster virus.

**Table 2:** Comparison of bleb morphology based on the Indiana Bleb Appearance Grading Scale at 1 month.

	Successful, *n* = 16 Eyes	Unsuccessful, *n* = 6 Eyes	*p*-Value *
Bleb height			>0.999
H0: flat bleb			
H1: low bleb			
H2: medium bleb	16 (100%)	6 (100%)	
H3: high bleb			
Horizontal extent			0.094
E0: <1 clock hours			
E1: ≥1 <2 clock hours		1 (16.7%)	
E2: ≥2 <4 clock hours	10 (62.5%)	5 (83.3%)	
E3: ≥4 clock hours	6 (37.5%)		
Vascularity			>0.999
V0: avascular white			
V1: avascular cystic			
V2: mild vascularity	15 (93.8%)	6 (100%)	
V3: moderate vascularity	1 (6.3%)		
V4: extensive vascularity			
Seidel test			>0.999
S0: no leak	16 (100%)	6 (100%)	
S1: multiple pinpoint leaks			
S2: streaming leak			

Values are presented as number (%). * Wilcoxon signed-rank test.

**Table 3:** Early intrableb parameters at 1 month assessed with anterior segment optical coherence tomography after trabeculectomy according to surgical success at 12 months.

Intrableb Parameters	Entire, *n* = 22	Successful, *n* = 16	Unsuccessful, *n* = 6	*p*-Value *
Bleb height, μm	1523.57 ± 181.06	1555.38 ± 178.58	1438.75 ± 173.61	0.178
Bleb wall thickness, μm	813.02 ± 401.35	898.47 ± 330.74	585.17 ± 512.55	**0.033**
Striping layer thickness, μm	376.14 ± 338.67	431.81 ± 349.96	227.67 ± 279.19	0.115
Striping/Bleb wall ratio	0.42 ± 0.24	0.44 ± 0.20	0.37 ± 0.35	0.407
Bleb wall reflectivity	100.36 ± 25.60	91.53 ± 18.90	123.88 ± 27.71	**0.021**
Fluid-filled space area, mm^2^	2.81 ± 1.50	2.70 ± 1.27	3.10 ± 2.11	0.541
Fluid-filled space height, μm	710.55 ± 331.50	656.91 ± 275.26	853.58 ± 447.76	0.083
Microcyst formation	16 (72.7)	14 (87.5)	2 (33.3)	**0.025**

Values are presented as mean ± standard deviation or number (%), unless otherwise indicated. * Comparison between successful and unsuccessful eyes. The significant differences between the two groups are marked in bold.

**Table 4:** Early intrableb parameters associated with successful IOP control determined with stepwise logistic regression analyses.

	Univariate		Multivariate Model	
Prognostic Factor	HR (95% CI)	*p*-Value	HR (95% CI)	*p*-Value
Sex (reference male)	0.333 (0.031, 3.579)	0.361		
Age	0.902 (0.788, 1.034)	0.139		
Glaucoma type (reference uveitic)	2.571 (0.361, 18.326)	0.346		
Preoperative lens status (reference phakic)	0.778 (0.119, 5.110)	0.793		
Number of preoperative glaucoma medication	1.908 (0.317, 11.474)	0.480		
Axial length	0.935 (0.572, 1.530)	0.790		
Preoperative visual acuity (logMAR)	1.234 (0.457, 3.330)	0.679		
Preoperative mean deviation	1.060 (0.931, 1.206)	0.380		
Preoperative IOP	0.935 (0.841, 1.039)	0.210		
Postoperative IOP at AS-OCT test (1 month)	1.443 (0.954, 2.183)	0.083		
Intrableb parameters at 1 month				
Bleb height	0.996 (0.991, 1.002)	0.184		
Bleb wall thickness	0.997 (0.994, 1.001)	0.121		
Striping layer thickness	0.997 (0.992, 1.002)	0.229		
Striping/Bleb wall ratio	0.292 (0.005, 18.241)	0.559		
Bleb wall reflectivity	1.072 (1.006, 1.141)	0.032	1.072 (1.006, 1.141)	0.032
Fluid-filled space area	1.214 (0.627, 2.351)	0.566		
Fluid-filled space height	1.002 (0.999, 1.006)	0.224		
Microcyst formation	0.071 (0.008, 0.680)	0.022		

## Data Availability

The data that support the findings of this study are not publicly available because they contain information that could compromise the privacy of research participants but are available from the corresponding author (S.M.) upon reasonable request.

## Supplementary Materials

The following supporting information can be downloaded at: https://www.mdpi.com/article/10.3390/jcm14155499/s1, Table S1: Early intrableb parameters at 1 month assessed with anterior-segment optical coherence tomography after trabeculectomy in successful eyes between uveitic and neovascular glaucoma.
