# Supplementary material for: Early Intrableb Features on Anterior Segment Swept-Source Optical Coherence Tomography Predict Surgical Success After Trabeculectomy in Uveitic and Neovascular Glaucoma
Source: J Clin Med. 2025 Aug 5;14(15):5499. doi: 10.3390/jcm14155499 (PMC12347853; doi:10.3390/jcm14155499)
Supplement: Supplementary file 1 [file jcm-14-05499-s001.zip › jcm-3786172-supplementary.pdf]

Supplementary Table S1. Early intrableb parameters at 1 month assessed with anterior-segment optical coherence tomography after trabeculectomy in successful eyes between uveitic and neovascular glaucoma

Intrableb parameters	Successful, n = 16	UG, n = 9	NVG, n = 7	p-value*
Bleb height, µm	1555.38 ± 178.58	1595.94 ± 175.02	1503.21 ± 182.32	0.351
Bleb wall thickness, µm	898.47 ± 330.74	979.94 ± .377.50	793.71 ± 246.38	0.351
Striping layer thickness, µm	431.81 ± 349.96	516.28 ± 434.82	323.21 ± 172.12	0.536
Striping/Bleb wall ratio	0.44 ± 0.20	0.47 ± 0.24	0.39 ± 0.13	0.681
Bleb wall reflectivity	91.53 ± 18.90	88.70 ± 16.70	95.18 ± 22.22	0.351
Fluid-filled space area, mm^2	2.70 ± 1.27	2.41 ± 1.45	3.07 ± 0.96	0.408
Fluid-filled space height, µm	656.91 ± 275.26	616.00 ± 326.03	709.50 ± 204.85	0.758
Microcyst formation	14 (87.5)	7 (77.8)	7 (100)	0.475

Values are presented as mean ± standard deviation or number (%) unless otherwise indicated. *Comparison between the two successful groups. UG, uveitic glaucoma; NVG, neovascular glaucoma
